# The Diagnosis of Malignant Endocarditis

**Published:** 1899-01-14

**Authors:** F. Percival Mackie

**Affiliations:** Assistant Medical Officer, County Asylum, Shrewsbury; late Assistant House Physician at Bristol General Hospital.


### HOSPITAL CLINICS AND MEDICAL PROGRESS.

### THE DIAGNOSIS OF MALIGNANT ENDOCARDITIS.

By F. PERCIVAL MACKIE, L.R.C.P.Lond., M.R.C.S.Eng., Assistant Medical Officer, County Asylum, Shrewsbury; late Assistant House Physician at Bristol General Hospital.

THE fact that this disease so closely simulates some others that it is consequently not infrequently overlooked till late in its course, and, further, that it is almost always fatal, makes it the more important that we should fully recognise its protean nature, and always consider the possibility of its existence in any obscure pyrexial condition.

Few diseases vary so greatly in the clinical pictures they present; any condition, from that of an almost chronic endocarditis lasting many months on the one hand, to that of a most intense blood-poisoning lasting only a few days on the other, may be represented, yet all forms are almost certainly fatal. The degree of acuteness, and also to some extent the type of the disease, depends upon the local endocardial condition more than any other one factor.

From a pathological point of view three kinds are recognised, viz, the vegetative, the ulcerative, and the suppurative. They are not generally met with purely as such, but in association, with one or other process predominating. It is possible to some extent to estimate the character and degree of the morbid change existing in the endocardium by the type of the disease. Thus the vegetative form is generally associated with embolic phenomena and with marked cardiac signs in the direction of murmurs, and is therefore the least likely of all forms to be overlooked, unless, as before pointed out, it be engrafted upon old valvular disease, when its presence may not be suspected till late in its course. The ulcerative form usually gives rise to marked toxic symptoms. Emboli may be set free by the gradual nibbling away of small pieces of the valve; these, however, are not generally large, and therefore do not give rise to such marked symptoms as result from the detachment of large masses of septic fibrin in the vegetative form.

I believe that those cases which present neither marked heart mischief nor yet embolic phenomena, but approach more nearly the typhoid type, will usually be found to be associated with ulcerative change, without much vegetation and without abscess formation, and that it is just this class of case which is the most difficult to diagnose. The third variety, the suppurative, is most commonly associated with the septic or pyæmic type. The emboli, if any are formed, are caused by the rupture of an endocardial abscess, which may occasion multiple infarction of various organs, and, if plugging the terminal cutaneous arterioles, giving rise to a papular rash during life. Rigors accompany the discharge into the blood of any fresh septic collection.

As regards the causation of these morbid changes the presence of virulent micro-organisms is the all-important factor. I say virulent advisedly, as organisms are found also in simple endocarditis, their effects depending not on their morphology, because in this point they are identi[illegible] but on their toxicity, which means the net difference been the virulence of the invader and the resistance of the invaded. After the careful consideration of any case it may be impossible to indicate the source of infection, just as it may be in pyæmia, tetanus, and other microbic diseases, and the word "idiopathic" used in this connection should be read to mean ignorance as regards origin, as it is obvious that no micro-organismal disease can possibly arise *de novo*.

It may be advantageous to indicate the various types the disease may assume.

To take the most chronic type. A person known to suffer from chronic valvular disease may, after an indefinite febrile attack or after a strain, develop cardiac irregularity and irritability, with varying pyrexia and perhaps an occasional rigor. Such a state of affairs may go on for several months, and the rest in bed may cause some improvement, so that the former diagnosis, if such were made, may be doubted. If there were murmurs known to exist before, the occurrence of fresh ones might be prevented, or anyhow the soft bruits of malignant endocarditis would be drowned by the harsher ones dependent upon old valvular disease. If, in addition to this, there are no embolic phenomena, as will sometimes be the case in the more or less chronic condition existing, two great factors in the diagnosis will be absent. In such a case the persistent pyrexia, increasing feebleness or irregularity of the cardiac action, together with the steady progress of the disease, will be the chief factors to aid us in coming to a conclusion; yet a certain diagnosis may not be arrived at till a short time before death, notwithstanding the fact that the disease has been existent for some months.

Another group presenting marked cardiac signs occurs in the course of rheumatic fever, and runs a more acute course than the last. To take an actual case which occurred: A. S., male, æt. 32, admitted to Bristol General Hospital, was found to have an ordinary attack of acute rheumatism, with local manifestation in one knee-joint, which disappeared and came in the ankle; a temperature varying between 100 and 103 F., and a soft mitral murmur during systole. His temperature went down, and his joint and muscular troubles disappeared under salicylates, and he was about to be allowed up, when, without any return of pain, the temperature began to go up, and a soft murmur appeared at the aortic orifice. The temperature persisted, and the two murmurs varied in intensity and in tone from time to time, and the patient got steadily worse. After six weeks of this he had a rigor, and went rapidly down-hill, developing rigors, sweats, and infarctions before death, and died in coma nine weeks after admission.

In some cases there may be no cardiac symptoms, but everything points to some other organ; in this case the lung. A man, æt. 36, of drunken habits, was admitted for commencing pneumonia of left lung, which showed signs of partial consolidation at the base. Instead of clearing up, it resolved partially and wandered about, presenting consolidation in several patches simultaneously, and attempted resolution in parts previously consolidated; in short, a condition of lobular pneumonia.

The temperature was markedly irregular; there were no murmurs, rigors, or sweats. He was considered to have a low pneumonia. The irregularity of its signs was explained by his dissolute life, and also by the probability of its being modified by a recent attack of influenza. He, however, got suddenly worse, had a very high temperature, rigor, sweating, and signs of pus in the right shoulder-joint. A mitral systolic murmur appeared 24 hours before death. The autopsy showed that the endocardial disease had probably been primary, and the lung secondary; yet, despite the fact that the mitral valve was extensively ulcerated, there was absolutely no evidence of it until a few hours before death.

Though this was the order of events in this case, it must be remembered that pneumonia is of all the most frequent antecedent of malignant endocarditis, and a not infrequent type of case is the following: A patient, who has passed through a typical attack of acute pneumonia, is on the way towards convalescence when the temperature begins to oscillate; subsequently cardiac excitement and development of a murmur arouses suspicion, confirmed later by somnolence, sweats, and embolisms, going on to a typhoid state, in which death occurs.

*(To be continued.)*

